# Speed breeding short-day crops by LED-controlled light schemes

**DOI:** 10.1007/s00122-020-03601-4

**Published:** 2020-05-12

**Authors:** Felix Jähne, Volker Hahn, Tobias Würschum, Willmar L. Leiser

**Affiliations:** State Plant Breeding Institute, University of Hohenheim, Fruwirthstr. 21, 70599 Stuttgart, Germany

## Abstract

**Key message:**

A simple and rapid speed breeding system was developed for short-day crops that enables up to five generations per year using LED lighting systems that allow very specific adjustments regarding light intensity and quality.

**Abstract:**

Plant breeding is a key element for future agricultural production that needs to cope with a growing human population and climate change. However, the process of developing suitable cultivars is time-consuming, not least because of the long generation times of crops. Recently, speed breeding has been introduced for long-day crops, but a similar protocol for short-day crops is lacking to date. In this study, we present a speed breeding protocol based on light-emitting diodes (LEDs) that allow to modify light quality, and exemplarily demonstrate its effectiveness for the short-day crops soybean (*Glycine max*), rice (*Oryza sativa*) and amaranth (*Amaranthus* spp.). Adjusting the photoperiod to 10 h and using a blue-light enriched, far-red-deprived light spectrum facilitated the growth of short and sturdy soybean plants that flowered ~ 23 days after sowing and matured within 77 days, thus allowing up to five generations per year. In rice and amaranth, flowering was achieved ~ 60 and ~ 35 days after sowing, respectively. Interestingly, the use of far-red light advanced flowering by 10 and 20 days in some amaranth and rice genotypes, respectively, but had no impact on flowering in soybeans, highlighting the importance of light quality for speed breeding protocols. Taken together, our short-day crops’ speed breeding protocol enables several generations per year using crop-specific LED-based lighting regimes, without the need of tissue culture tools such as embryo rescue. Moreover, this approach can be readily applied to a multi-storey 96-cell tray-based system to integrate speed breeding with genomics, toward a higher improvement rate in breeding.

**Electronic supplementary material:**

The online version of this article (10.1007/s00122-020-03601-4) contains supplementary material, which is available to authorized users.

## Introduction

Key message: A simple and rapid speed breeding system was developed for short-day crops that enables up to five generations per year using LED lighting systems that allow very specific adjustments regarding light intensity and quality.

**Fig. 1:**
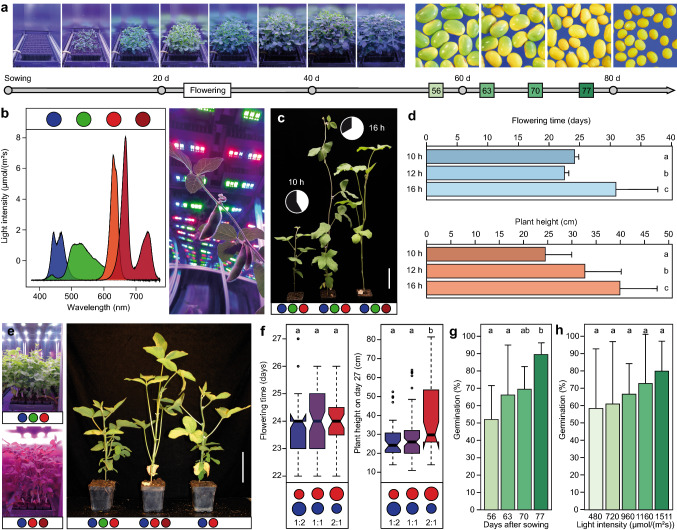
Development of a speed breeding protocol for soybean. **a** Schematic overview of the steps to be optimized: the time to flowering and to maturity. **b** LED light spectrum with four separately controllable channels and impression from inside a LED speed breeding box. **c** Soybean grown under short-day and long-day conditions. Scale bar = 10 cm. **d** Bar plots showing the effects of day length on flowering time and plant height under blue, green and red light conditions. **e** Effect of red and far-red light on plant morphology. Scale bar = 10 cm. **f** Flowering time and plant height dependent on different ratios between red and blue light. **g** Germination rate of soybean seeds harvested at different time points. **h** Effect of light intensity on germination rate at 63 days after sowing

Conventional breeding of new and improved cultivars can take up to 12 years for annual crops from the point of crossing parental material until commercial release of novel cultivars. It is possible to significantly shorten this long and tedious process, for example by the use of winter nurseries, utilization of the doubled haploid technique (Thomas and Forster 2003) or the use of genetic engineering or genome editing (Araki and Ishii 2015). However, these approaches have severe disadvantages: winter nurseries are often expensive, logistically complicated to manage and do not guarantee successful seed production; doubled haploids are not available for most crops and often require highly qualified personnel and financial resources; and transgenic or genome-edited crops are often not a viable option because of political legislation or societal skepticism. Speed breeding was recently proposed by Watson et al. (2018) as an alternative to facilitate the simple and fast generation of new crop cultivars. The proposed speed breeding protocol reduces the generation time of long-day crops by an extension of the photoperiod to almost full day and harvest of immature seeds. However, this approach is limited to long-day crops and cannot be applied to short-day and photoperiod-sensitive crops, such as the globally important soybean and rice, because the prolonged photoperiod will prevent their flowering. Furthermore, the proposed protocol does not consider light quality to optimize the speed breeding procedure. Here, we present a light-emitting diode (LED)-based speed breeding protocol for short-day crops, that in addition to photoperiod also highlights the effect of light quality parameters for a practicable and high-throughput rapid single seed descent (rSSD) system (Fig. 1a, b).

## Material and methods

### Plant material and growth conditions

For the soybean experiments, we used seven commercial European soybean cultivars and one soybean line from the US Department of Agriculture Gene Bank. The panel of genotypes represents a broad range of maturity groups, from very early, photoperiod-insensitive cultivars that can be grown in Central Europe (maturity group G000), to late and photoperiod-sensitive subtropical lines (maturity group G5). Table S1 gives an overview about this panel, including cultivar name, maturity group and allelic constitution at the major maturity loci *E1*, *E2*, *E3* and *E4*. The rice and amaranth flowering time experiments included seven subtropical and tropical rice lines and seven amaranth lines with varying photoperiodism, the names of which can be found in Tables S2 and S3.

A mixture of two parts turf/wood fiber/clay granule substrate (NPKMgS 180/210/360/100/150 mg/l, Substrate 5, Klasmann-Deilmann GmbH) and one part sterilized soil substrate (compost soil/sand 3:1) was used as growth medium for all experiments. Substrate mixture for soybean experiments was inoculated with ‘Soya Bean Inoculum’ from Legume Technology Ltd. (Nottinghamshire) to allow formation of rhizobia. Plants were grown in pots measuring 7 cm × 7 cm × 8 cm (40 pots per tray) and for a few experiments in 96-cell plates (pot size 2 cm × 2 cm × 8 cm each). Watering was adjusted according to the plants’ developmental status which in the soybean experiments required a weekly adaptation until pod onset. After pod onset, the water consumption of the plants remained constant at ~ 2 l/day on a tray with 40 plants. We watered the plants from below into the trays once every day with an automatic watering system.

All experiments were performed within a climate-controlled chamber. The temperature was set to 28 °C day and night. Humidity ranged from 80% to almost 100%, depending on the number of experiments in the climate chamber and watering regime.

### Speed breeding boxes

Inside the climate chamber, we installed 12 different speed breeding boxes (80 cm × 60 cm × 120 cm) that allowed a parallel testing of different settings for parameter optimization (Fig. S1). The boxes were set up with reflecting surfaces on the inside to ensure optimal light usage. The light sources in each speed breeding box were light-emitting diodes (LEDs), which have the advantageous ability to emit very wavelength-specific light spectra as opposed to incandescent lamps. Several different LED systems were explored for all our 66 experiments using soybean, rice and amaranth:

#### LED modules: Ecotune, Daypro and Beaglebone

CompLED (COMPLED Solutions GmbH, Dresden, Germany) designed these LED modules. Hitz et al. (2019a, b) described the technical specifics of these speed breeding boxes in detail. Different types of LEDs were combined in four to six channels in each growth module. These channels are independently accessible via a user interface, which allows to regulate each channel in its intensity of light emission and the time of light exposure. The light emission intensities, and the spectra of each channel as well as all channels combined are shown for each module at 100% light intensity in Table S4 and Fig. S2 (1–3).

#### LED module: Relumity

Relumity (Relumity TTI-Technologie-Transfer-Initiative GmbH, Stuttgart, Germany) builds two LED modules of this type. Contrary to other manufacturers, Relumity uses a modular setup, meaning that LEDs of the same wavelength are spotted on separate circuit boards, making it easy to alter light regimes by installing different circuit boards. The light recipe in this chamber type is controlled manually, the day length automatically by timer. The light emission intensities, and the spectra of each channel and all channels combined are shown at 100% light intensity in Table S4 and Fig. S2 (4 and 5).

#### LED module: Blue panel and blue rail

Growking (Growking.de, Leinfelden-Echterdingen, Germany) manufactured a third LED module in two different light intensities. Light regimes and intensities were not adjustable after delivery, but both LED systems were designed according to our requests. The light emission intensities and spectra of the Growking Blue Panel and Growking Blue Rail are shown in Table S4 and Fig. S2.

### Rice and amaranth experiments

For the experiments with rice and amaranth, we used the following light protocol in the ‘*ecotune*’ LED growth chambers: 556 and 574 µmol/(m^2^s) light intensity, respectively, 10-h light and 14-h night, near-red light recipe with channels 1 + 2 + 3 and far-red light recipe with channels 1 + 2 + 4 (Table S4).

### Phenotyping flowering and plant height

The soybean experiments were performed with five replicates of each cultivar. The 40 pots were completely randomized on each tray. For the experiments with staggered harvest dates, we used the three cultivars ‘ES Senator’, ‘Josefine’ and ‘Nogoshi’ and divided each tray into four compartments with 10 plants each (minimum three replicates per genotype per compartment) as shown exemplary in Fig. S4. We examined the plants’ flowering every day after initiation of the budding phase. Flowering time was noted as days after planting upon appearance of the first petals on each plant. Since we aimed to present a rapid SSD protocol, we decided to stop every experiment in which a genotype exceeded a flowering time of 40 days. We measured plant height 20 and 27 days after sowing as the distance from the plant’s shoot apical meristem to the soil. Some genotypes showed indeterminate growth under these conditions and were pruned on day 30 after sowing. An overview of the performed experiments, light quality and quantity conditions, flowering and plant height per genotype and per treatment is shown in Table S5. We noted flowering time for rice when the first panicle emerged and for amaranth when the first pollen sacs were visible. Plant height of rice and amaranth was measured upon termination of flowering from the tip of the inflorescence to the soil. Tukey's honest significant difference (HSD) test was used to test pairwise comparisons and significant differences of means (Tukey 1977). Different letters on the top of box plots and bar plots indicate significant differences of the means according to Tukey’s HSD test (*α* = 0.05).

### Harvesting

Harvesting dates (~ 63 and ~ 70 days) differed between the performed experiments in order to test different maturity periods. In all experiments, watering was ceased 5 days before harvesting date to accelerate the ripening process in seeds. In experiments with staggered harvest dates (55/56, 63, 70 and 77 days), we moved all plants that reached the end of the watering period into a smaller waterproof compartment tray in order to prevent those plants from being watered for 5 days (Fig. S3). On the harvest day, the pods of every genotype were bulked. Afterward, a drying treatment was applied for 24 h at 37 °C to facilitate manual cleanup of the seeds from their hulls. Table S6 shows a list of all experiments that were carried out until the harvest of the plants to assess their germination ability.

### Germination experiments

Experiments concerning germination tests started with the following conditions: blue, green and red (630 nm) lights under short-day conditions (10 h) with a light intensity of 556 µmol/m^2^ s until day 35 after planting (Table S5; Exp. 29, 33, 36, 37, 40, 46 and 53), and were then transferred to different conditions until harvest (Table S6). Exceptions from this procedure were made in experiments 51, 55, 56 and 66 in which the plants were grown under the same conditions from germination to harvest (see corresponding Exp. 43, 48, 50 and 61 in Table S5). All seeds were treated with 0.05% thiram solution (Aatiram 65, Cheminova) for two minutes in order to reduce fungal growth during the germination tests. Germination took place on filter paper; under dark conditions, the temperature was set to 25 °C. Sterile water was used to keep the filter paper moist. Germinated seeds were counted on day 7. Missing radicle protrusion was considered as not germinated. Germination rate was calculated as the quotient of germinated seeds divided by the total number of used seeds in the experiment. In order to investigate the impact of gibberellin on the germination of seeds from 56-day-old soybean plants, we used the genotypes ‘Merlin,’ ‘ES Senator,’ ‘Amphor’ and ‘Aires.’ The plants were grown at a light intensity of 420 µmol/m^2^ s without far-red light. Germination protocol was as described above, with one half of the laid-out seeds per genotype watered with ddH_2_0 and the other half with 0.1% (v/v) Gibb + -Solution (Gibb^Plus^, Plantan). Germinated seeds were counted on day 4, 7 and 10 after the start of the experiment.

## Results

In photoperiod-sensitive short-day crops, long-day conditions hinder the initiation of flowering. Hence, lighting protocols longer than 12 h can be expected to lead to delayed flowering, but on the other hand may enhance carbon accumulation and might therefore speed up seed production (Chatterton and Silvius 1979; Jensen and Veierskov 1998). In addition, it is known that phytochrome-deficient genotypes of rice, sorghum and soybean flower earlier under long-day conditions (Izawa et al. 2000; Childs et al. 1997; Tsubokura et al. 2013; Cober et al. 1996) and that far-red light promotes the transformation of active into inactive phytochromes (Carré et al. 2005). Focusing on soybean, we therefore first evaluated light regimes with an increased light period of ≥ 12 h, but combined with a low red to far-red ratio (< 700 nm: > 700 nm) to potentially achieve early flowering with an increased photosynthesis rate (Table S5). In order to enhance photosynthesis rate further during night conditions, blue light (450–490 nm) was enabled. Smith and Whitelam (1997), Childs et al. (1997) and Craig and Runkle (2013) suggested that far-red lighting leads to earlier flowering. In contrast to these previous findings, in our experiments neither far-red nor an additional blue light treatment at night could accelerate flowering of the soybean genotypes. In most cases, average flowering time surpassed four weeks and was highly heterogeneous among the soybean genotypes, showing the different responses to day length of cultivars from different maturity groups. Generally, time to flowering was hastened and the plants were shorter under ≤ 12 h day length protocols (Fig. 1c, d). As shorter day length led to faster flowering, short-day protocols with light exposures of 12, 10 or 8 h were examined, which reduced the average flowering time to 23.9 ± 1.8, 23.7 ± 1.4 or 24.0 ± 0.8 days after planting, respectively. Moreover, under such conditions all soybean genotypes flowered in a homogeneous fashion, allowing to use the system for early and late maturity groups in the same growing cycle (Fig. S4).

Next, several experiments were performed under a day length set to 10 h, to determine the optimal light regime that maintains early flowering while reducing plant height so that the plants would fit into multi-storey tray systems. Far-red light (> 700 nm) did not affect flowering time, but led to an unwanted plant morphology with severely elongated petioles and consequently lodging, as also reported by Smith and Whitelam (1997), Franklin and Whitelam (2005) and Hitz et al. (2019a) (Fig. 1e). Hence, far-red lighting must be avoided to obtain sturdy soybean plants suitable for compact high-throughput systems. Avoiding far-red wavelengths, we evaluated the impact of different red/blue light ratios (Fig. 1f). Generally, a lower red/blue light ratio led to shorter and sturdier plants without affecting flowering time, but when using only blue light no further height reduction could be observed. Although green light (500–560 nm) did not greatly affect flowering time and plant height of soybean, visual evaluation of the plants (scoring of diseases, presence of pests) was highly improved by including green light into the lighting protocol (Fig. 1e). Thus, a multi-storey speed breeding protocol for soybean must avoid far-red light (> 700 nm) and should have a low red/blue light ratio, with green or cool white LEDs (4000 K) included for optimal visual observations.

Since lighting schemes with more than 12-h light are not suitable for short-day crops, but as enhanced photosynthesis rates may shorten cycle durations, we evaluated the impact of light intensity on flowering time and plant morphology for a 10-h lighting scheme. Increasing light intensity above 1000 µmol/m^2^ s led to a ~ 2 days earlier flowering (21.8 ± 1.1 vs. 23.9 ± 1.1 for light intensity of 500–900 µmol/m^2^ s) and shorter plant stature. These findings point to a further speed-up option by increasing light intensity, but these subtle changes will come with additional expenses due to higher energy costs and more expensive LED systems. Thus, for a well operating system a light intensity of ~ 500 µmol/m^2^ s at 50 cm from the light source should suffice to achieve fast generation times on a moderate budget.

**Fig. 2:**
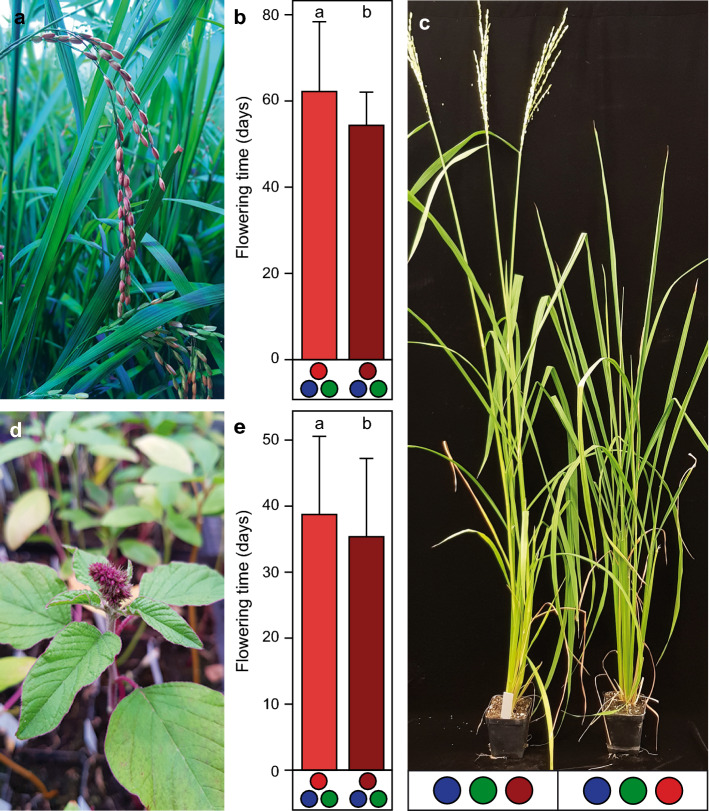
Speed breeding rice and amaranth. **a** Rice and **d** amaranth flowering under speed breeding conditions. **b** Effect of red and far-red light on flowering time of rice. **e** Effect of red and far-red light on flowering time of amaranth. **c** Rice genotype ‘Nerica L-19’ on day 70 after sowing under far-red and red light conditions

We next investigated whether this optimized light protocol elaborated for soybean would also enable speed breeding of other short-day crops, and ran flowering time experiments with rice and amaranth. In addition, we again assessed the impact of far-red light on flowering time and plant morphology. Contrary to soybean, rice flowered on average 7.9 days earlier under far-red light conditions than under far-red deprived lighting schemes (54.3 ± 7.7 vs. 62.2 ± 16.2 days) (Fig. 2a–c; Table S7). In fact, the majority of the genotypes flowered almost equally fast under both light conditions. However, two of the genotypes which took the longest to reach inflorescence stage (‘Primavera’ and ‘Nerica L-19′) flowered 20 days earlier under far-red light compared to the near-red light protocol. A similar picture was obtained for amaranth that flowered on average ~ 3 days earlier under far-red light (35.35 ± 11.87 vs. 38.71 ± 11.85) (Fig. 2d, e; Table S8). As observed for rice, there was a strong genotypic dependency on light quality (Fig. S5, Table S7, S8). Similar to soybean, plant height of rice and amaranth increased under far-red conditions, but did not lead to instable plant stands as observed for soybean. Generally, these findings show that speed breeding protocols cannot be readily interchanged from one short-day crop to another and especially that light quality must be considered for an optimized genotype-independent speed breeding system.

Achieving fast flowering is the first step in setting up speed breeding protocols. Once fast flowering is reached, fast maturity or germination of immature seeds needs to be accomplished. Aiming at a two-month seed-to-seed protocol for soybean, we evaluated germination rates at staggered harvest time points (day 56, 63, 70 and 77). Mean germination increased from ~ 50% on day 56 to ~ 90% on day 77, and likewise the homogeneity of germination across the genotypes increased (Fig. 1g; Fig. S6). Although 77 days after sowing yielded very good germination rates, we tested whether increased light intensities could further improve germination rate at earlier time points, by either using stronger light sources or extending lighting duration after flowering onset. We observed a general increase in germination rate with lighting intensity, i.e., using 10-h day lengths from ~ 60% at 480 µmol/m^2^ s to ~ 80% at 1511 µmol/m^2^ s at 63 days after sowing (Fig. 1h). To further speed up the maturation process, day length was extended from 10 to 18 h on day 35 after pod onset was initiated. Although germination rate increased in the 18-h treatment by ~ 8% at 63 days after sowing, germination rate was still < 70% and highly variable among genotypes. Next, we increased the light intensity to 1160 µmol/m^2^ s and aimed at a harvest time on day 63 after sowing with three different light regimes. Germination was slightly increased when including green light (78%) as compared to the red and blue (71%) or the far-red lighting schemes (62%). Although acceptable mean germination rates were achieved, there were still large genotypic differences as compared to seeds that matured until day 70. Furthermore, such high light intensities again come with the more expensive LED light sources and energy costs. Given that some genotypes showed optimal germination results even in the 63-day-long tests using lower light intensities, it is possible to reduce the generation time of certain populations or breeding material. However, since synchronization of maturity could not be guaranteed across genotypes, we conclude that a longer maturation time to ~ 75 days is necessary for a speed breeding system that can operate genotype independently and without the need of time- and work-intensive methods such as the growth on sterile medium or embryo rescue in order to facilitate germination of unripe seeds. We also investigated whether a gibberellin treatment was able to further improve the germination of unripe seeds as suggested by Hickey et al. (2019). Soybean seeds harvested from 56-day-old plants that were treated with Gibb + solution increased their germination on average by 7% as opposed to the water control (Fig. S7). However, the germination was generally low at that early stage, a wide variation among genotypes was observed, and the difference between the treatments was not significant at any time point. More importantly, the Gibb + treatment had the unfortunate effect of elongating the hypo- and epicotyls of the germinated soybean seedlings, which is a highly counterproductive attribute keeping in mind a multi-storey growth chamber with limited space.

Although we established the protocol with larger pots, we also transferred it to 96-cell plates, with pot sizes of 75cm^3^, allowing ~ 750 plants per cubic meter of space (Fig. S8). Soybean growth and flowering time were comparable to the described protocol, with plant height reaching ~ 34 cm on day 28 and flowering at ~ 24 days after planting at a light intensity of ~ 1000 µmol/m^2^ s, and a seed-to-seed turnover of ~ 75 days. Seed number per plant was reduced in the 96-cell system, but with on average more than five seeds per plant sufficient for an rSSD system. Having a 96-cell-based system for plant growth allows the speed breeding protocol to be readily integrated with genotyping systems that are all based on 96-well microtiter plates. This will allow routine single marker and genomic selection approaches during the speed breeding process and reduce the error rate due to the full conformity between growth and DNA analysis system.

## Discussion

**Fig. 3:**
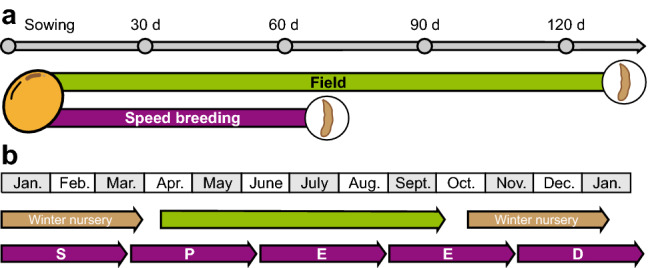
Speed breeding short-day crops. **a** Time required for one soybean generation under field conditions in central Europe compared to speed breeding. **b** In contrast to the field, where only one or with winter nursery 2–3 generations can be achieved per year, speed breeding enables five generations per year

Speed breeding is a powerful tool for plant breeding and plant genetic research. Our results for the three short-day crops, soybean, rice and amaranth, highlight the need for crop-specific lighting schemes that can speed up the time to flowering and maturity and might be utilized to improve germination. For soybean, we developed a protocol that allows up to five generations per year, as compared to one generation on the field or 2–3 generations if winter nurseries are used (Fig. 3). Increasing the speed of our protocol by raising the CO_2_ level can be considered (Nagatoshi and Fujita 2018), but might be only advantageous if a higher photosynthesis rate may also be guaranteed through more intense lighting. The additional speed should be weighed against the extra costs of the system setup and operational costs (e.g., energy).

In general, speed breeding enables the introgression of monogenic traits that are easily scored within the climate chamber, but also allows crosses between genotypes from different maturity groups, which may widen the genetic variation of the breeding material and hence enhances response to selection. By obtaining up to five generations per year in a speed breeding system will lead to an approximately doubled annual genetic gain as compared to a fast breeding program which uses winter nurseries. Furthermore, tools such as marker-assisted or genomic selection can easily be incorporated in a speed breeding system, since logistical hurdles, which may arise in winter nurseries, are circumvented.

The fast generation of homozygous lines not only allows to speed up workflows in practical plant breeding but also for research purposes. Owing to the specificity of the LEDs, this system can also be used to dissect the interaction of specific wavelengths and the plant’s physiological responses. Although our presented light protocol aims at researchers and breeders, companies working on and with indoor farming systems will profit from crop-specific research on light regimes like this one, too. Adjusted and smart lighting systems will play a pivotal role in urban farm production to meet future needs of the growing urban population that increasingly values short transportation routes, eco-friendly production and fresh food.

## Electronic supplementary material

Below is the link to the electronic supplementary material.

Supplementary file1 (DOCX 2642 kb)

Supplementary file2 (XLSX 65 kb)

Supplementary file3 (XLSX 32 kb)
