# Supplementary material for: Speed breeding short-day crops by LED-controlled light schemes
Source: Theor Appl Genet. 2020 May 12;133(8):2335–42. doi: 10.1007/s00122-020-03601-4 (PMC7360641; doi:10.1007/s00122-020-03601-4)
Supplement: Supplementary file 1 — Supplementary file1 (DOCX 2642 kb) [file 122_2020_3601_MOESM1_ESM.docx]

**Speed breeding short-day crops by LED-controlled light schemes**

Felix Jähne^1^, Volker Hahn^1^, Tobias Würschum^1^, Willmar L. Leiser^1^*

^1^State Plant Breeding Institute, University of Hohenheim, Fruwirthstr. 21, 70599 Stuttgart, Germany

* For correspondence (e-mail willmar_leiser@uni-hohenheim.de)

ORCID IDs: 0000-0003-4998-068X (FJ), 0000-0003-4837-1460 (VH), 0000-0002-7397-7731 (TW), 0000-0001-5964-5610 (WLL)

**Supplementary Files**

**Table S1** Overview of the soybean genotype set consisting of commercial European cultivars and a Japanese (USDA PI accession) line that were used for the soybean experiments.

| Cultivar | Maturity group | *E1* | *E2* | *E3* | *E4* | Country of Origin |
| --- | --- | --- | --- | --- | --- | --- |
| Merlin | G000 | *e1-nl* | *e2-ns* | *E3-Ha* | *e4-SORE1* | Canada |
| ESSenator | G000 | *e1-as* | *e2-ns* | *e3-tr* | *E4* | France |
| Amphor | G00 | *e1-nl* | *e2-ns* | *E3-Ha* | *e4-SORE1* | Austria |
| Josefine | G00 | *e1-nl* | *e2-ns* | *E3-Ha* | *E4* | Austria |
| Onix | G00 | *e1-as* | *e2-ns* | *E3-Ha* | *E4* | Romania |
| Aires | G0 | *e1-as* | *e2-ns* | *e3-tr* | *E4* | Italy |
| Sponsor | G2 | *e1-as* | *E2-in* | *e3-tr* | *E4* | Italy |
| Nogoshi | G5 | *E1* | *e2-ns* | *E3* | *E4* | Japan (USDA PI 200510) |

**Table S2** Overview of the used rice cultivars; Isc, inter-specific cross

| Cultivar | Species | Subspecies | | Type | County of origin |
| --- | --- | --- | --- | --- | --- |
|  |  | genetical | climatically |  |  |
| B22 | *O. sativa* | *japonica* | tropical | improved | Brazil |
| Chomrong | *O. sativa* | *japonica* | temperate | traditional | Nepal |
| CG14 | *O. glaberrima* | - | tropical | traditional | Senegal |
| Nerica 4 | Isc (*sat* × *gla*) |  |  | improved | Benin (Africa Rice Center) |
| Nerica L-19 | Isc (*sat* × *gla*) |  |  | improved | West Africa (Africa Rice Center) |
| Fofifa 172 | *O. sativa* | *japonica* | tropical | improved | Madagascar (FOFIFA) |
| Primavera | *O. sativa* | *japonica* | tropical | improved | Brazil |

**Table S3** Overview of the used amaranth genotypes.

| Genotype | Amaranth species | Accession Name | Country of origin |
| --- | --- | --- | --- |
| cau44 | *A. caudatus* | Ames 15178 | Argentina |
| hypo174 | *A. hypochondriacus* | PI 649623 | Mexico |
| quit185 | *A. quitensis* | PI 652426 | Brazil |
| quit189 | *A. quitensis* | PI 652422 | Brazil |
| hybr119 | *A. hybrid* | PI 604566 | Mexico |
| cau363 | *A. caudatus* | PI 511689 | Peru |
| hypo149 | *A. hypochondriacus* | Ames 5149 | Puerto Rico |

**Table S4** Summary of LED-light chambers used for experimentation.

| Chamber  type | Channel | Predominant  colour | Light intensity [µmol/(m²s)] at  100% power | Suppl.  Figure 7;  reference | Note |
| --- | --- | --- | --- | --- | --- |
| Ecotune | all | white | 778 (+22 UV) | 1a | sum intensity |
|  | 1 | blue | 130 | 1b |  |
|  | 2 | green | 222 | 1c |  |
|  | 3 | orange-red | 204 | 1d |  |
|  | 4 | red-far-red | 222 | 1e |  |
|  | UV | UV-a | 27 | / | not shown in grafic |
| Daypro | all | white | 830 (1050) | 3a | sum intensity, mirror surface in bracets |
|  | 1 | white | 289 (360) | 3b | mirror surface in bracets |
|  | 2 | blue | 102 (137) | 3c | mirror surface in bracets |
|  | 3 | red-far-red | 43 (58) | 3d | mirror surface in bracets |
|  | 4 | warm white | 99 (123) | 3e | mirror surface in bracets |
|  | 5 | cold white | 224 (284) | 3f | mirror surface in bracets |
|  | 6 | white | 78 (97) | 3g | mirror surface in bracets |
| Beaglebone | all | white | 2565 | 2a | sum intensity |
|  | 1 | blue | 477 | 2b |  |
|  | 2 | green | 218 | 2c |  |
|  | 3 | white | 438 | 2d |  |
|  | 4 | orange-red | 689 | 2e |  |
|  | 5 | red | 526 | 2f |  |
|  | 6 | far-red | 218 | 2g |  |
| Relumity 1 | all | purple | 880 | 4a | sum intensity |
|  | 1 | blue | 399 | 4b |  |
|  | 2 | orange | 57 | 4c |  |
|  | 3 | orange-red | 185 | 4d |  |
|  | 4 | red | 239 | 4e |  |
| Relumity 2 | all | white | 945 | 5a | sum intensity |
|  | 1 | blue | 399 | 5b |  |
|  | 2 | green | 122 | 5c |  |
|  | 3 | orange-red | 185 | 5d |  |
|  | 4 | red | 239 | 5e |  |
| Growking „Blue panel“ | / | cold white | 1511 | 6 |  |
| Growking „Blue rail“ | / | cold white | 493 | 7 |  |

**Table S5** Summary of soybean flowering experiments. Exp, experiment number; Cha, chamber type according to Table S4; Light, light quality recipe; Illu, illumination time in hours per day; Int, light intensity in µmol/(m²s); Geno, genotype name; Fl, mean flowering per genotype in days after planting; Sd, standard deviation; He20, mean plant height on day 20 in cm; He27, mean plant height on day 27 in cm; Fl exp, mean flowering time in days per experiment across all genotypes; He20 exp/He27 exp, mean plant height in cm per experiment across all genotypes on day 20/27.

Table S5 is provided as Excel file.

**Table S6** Summary of soybean germination experiments. Exp, experiment number; Cha, chamber type according to Table S4; Light, light quality recipe; Illu, illumination time in hours per day; Int, light intensity in µmol/(m²s); Geno, genotype name; Hd, harvest day; Seeds, number of harvested seeds per genotype; Ge, germination percentage per genotype; Ge exp, mean germination per experiment across all genotypes; Sd, standard deviation.

Table S6 is provided as Excel file.

**Table S7** Mean flowering time and plant height for each rice cultivar. FR, far-red treatment; R, only red and no far-red light; Sd, standard deviation.

| Rice genotype | Mean flowering time FR | Sd flowering time FR | Mean flowering time R | Sd flowering time R | Mean height FR | Sd height FR | Mean height R | Sd height FR |
| --- | --- | --- | --- | --- | --- | --- | --- | --- |
| Fofifa 172 | 45.40 | 3.29 | 46.20 | 4.92 | 79.40 | 10.50 | 74.40 | 17.77 |
| Chhomrong | 47.33 | 2.07 | 51.33 | 2.66 | 108.25 | 3.40 | 104.67 | 10.38 |
| B22 | 52.17 | 8.54 | 52.83 | 0.98 | 99.67 | 4.32 | 91.75 | 10.46 |
| Nerica 4 | 52.17 | 1.94 | 56.50 | 2.81 | 84.92 | 6.38 | 81.17 | 14.29 |
| CG14 | 54.00 | 7.68 | 58.20 | 2.49 | 106.40 | 7.57 | 104.50 | 10.98 |
| Primavera | 63.33 | 13.31 | 83.50 | 1.91 | 115.67 | 3.51 | 92.63 | 18.04 |
| Nerica L-19 | 66.00 | 7.21 | 86.83 | 2.93 | 120.67 | 7.26 | 104.33 | 16.95 |
|  |  |  |  |  |  |  |  |  |
| Mean | 54.34 | 7.70 | 62.2 | 16.18 | 102.14 | 15.28 | 93.35 | 12.14 |

**Table S8** Mean flowering time and plant height for each amaranth genotype. FR, far-red treatment; R, only red and no far-red light; Sd, standard deviation.

| Amaranth genotype | Mean flowering time FR | Sd flowering time FR | Mean flowering time R | Sd flowering time R | Mean height FR | Sd height FR | Mean height R | Sd height FR |
| --- | --- | --- | --- | --- | --- | --- | --- | --- |
| Cau44 | 24.00 | 0.00 | 24.00 | 0.00 | 23.25 | 5.50 | 28.25 | 1.50 |
| Hypo174 | 27.00 | 0.00 | 32.00 | 3.46 | 44.75 | 3.20 | 48.50 | 5.92 |
| Quit185 | 29.75 | 1.50 | 33.50 | 1.73 | 24.25 | 9.11 | 20.00 | 3.56 |
| Quit189 | 34.00 | 6.16 | 33.50 | 1.73 | 31.00 | 6.98 | 23.00 | 3.74 |
| Hybr119 | 35.00 | 0.00 | 47.00 | 3.27 | 38.50 | 3.87 | 30.50 | 2.38 |
| Cau363 | 37.67 | 5.51 | 41.00 | 5.19 | 24.33 | 6.35 | 19.33 | 2.08 |
| Hypo149 | 60.00 | 0.00 | 60.00 | 0.00 | 18.75 | 4.03 | 15.25 | 2.50 |
|  |  |  |  |  |  |  |  |  |
| Mean | 35.35 | 11.87 | 38.71 | 11.85 | 29.26 | 9.35 | 26.40 | 11.07 |


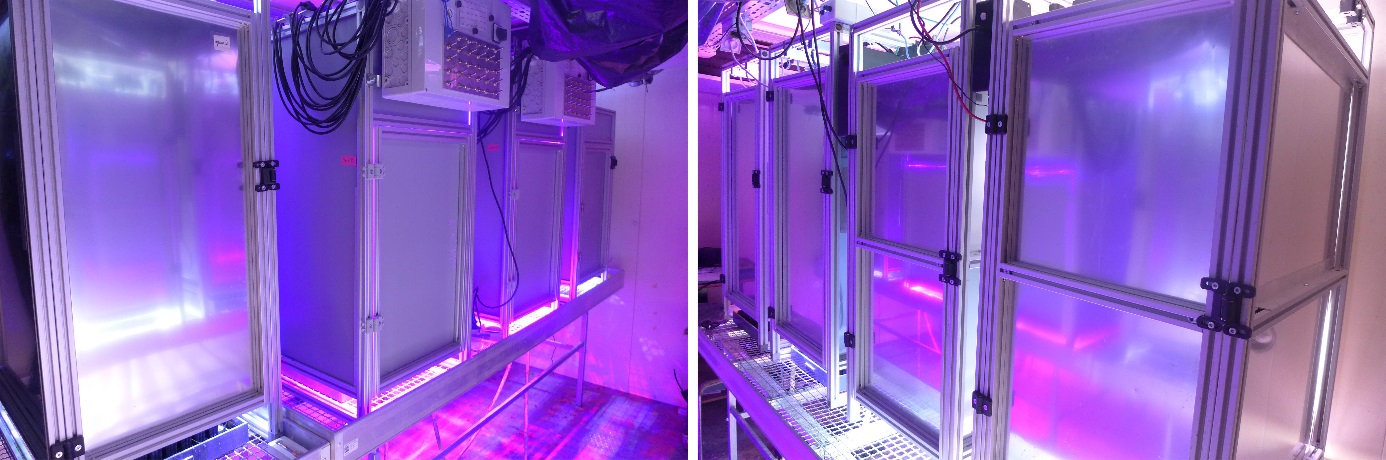


**Fig. S1** Speed-breeding boxes used for parallel testing of different settings for parameter optimization.


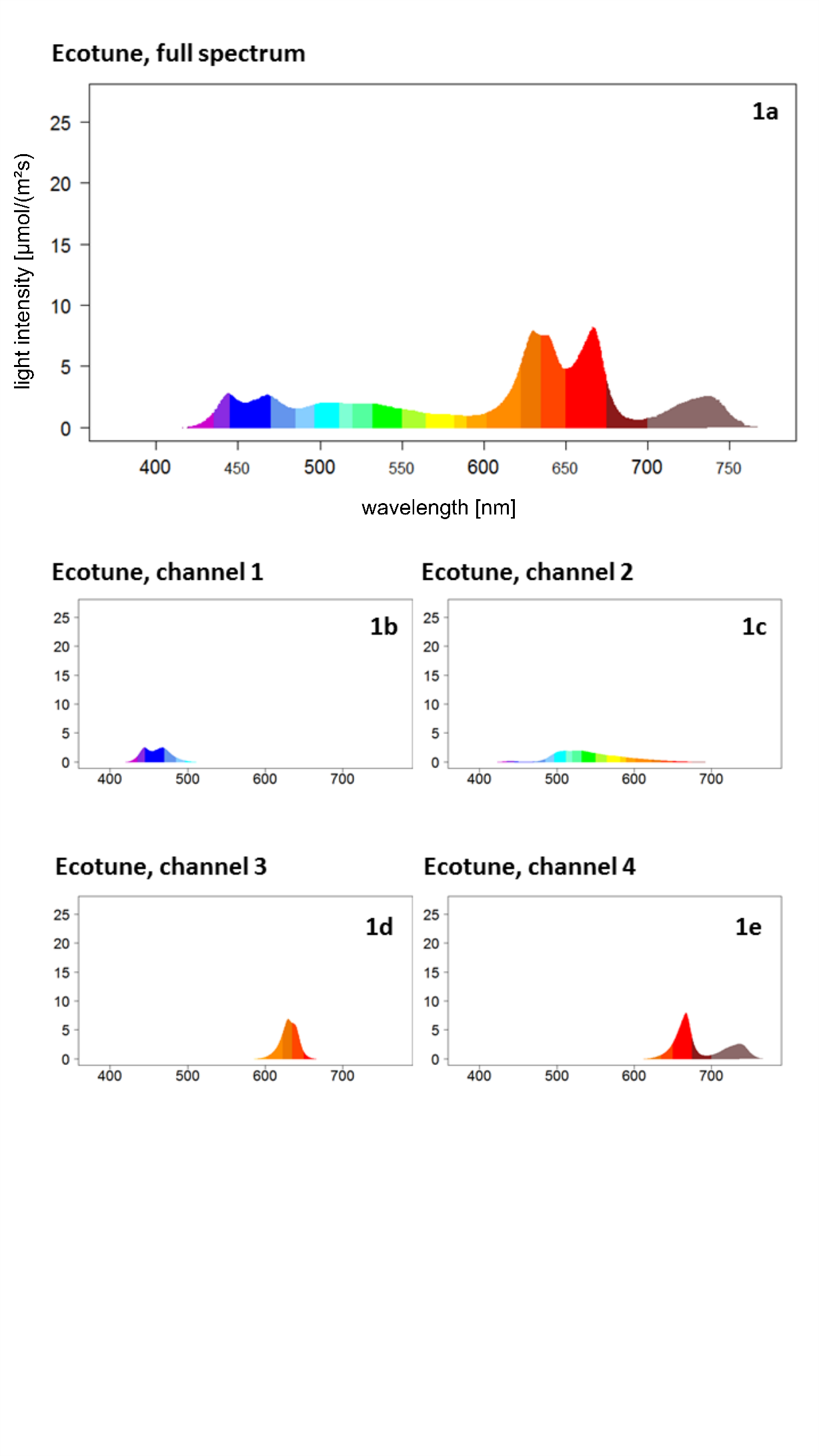


**Fig. S2.1** Emission spectrum of LED chamber type “Ecotone”. **a** All channels at 100% intensity combined; **b-e** single channels at 100%. Numbers 1a – 1e in the top right corner refer to Table S4.


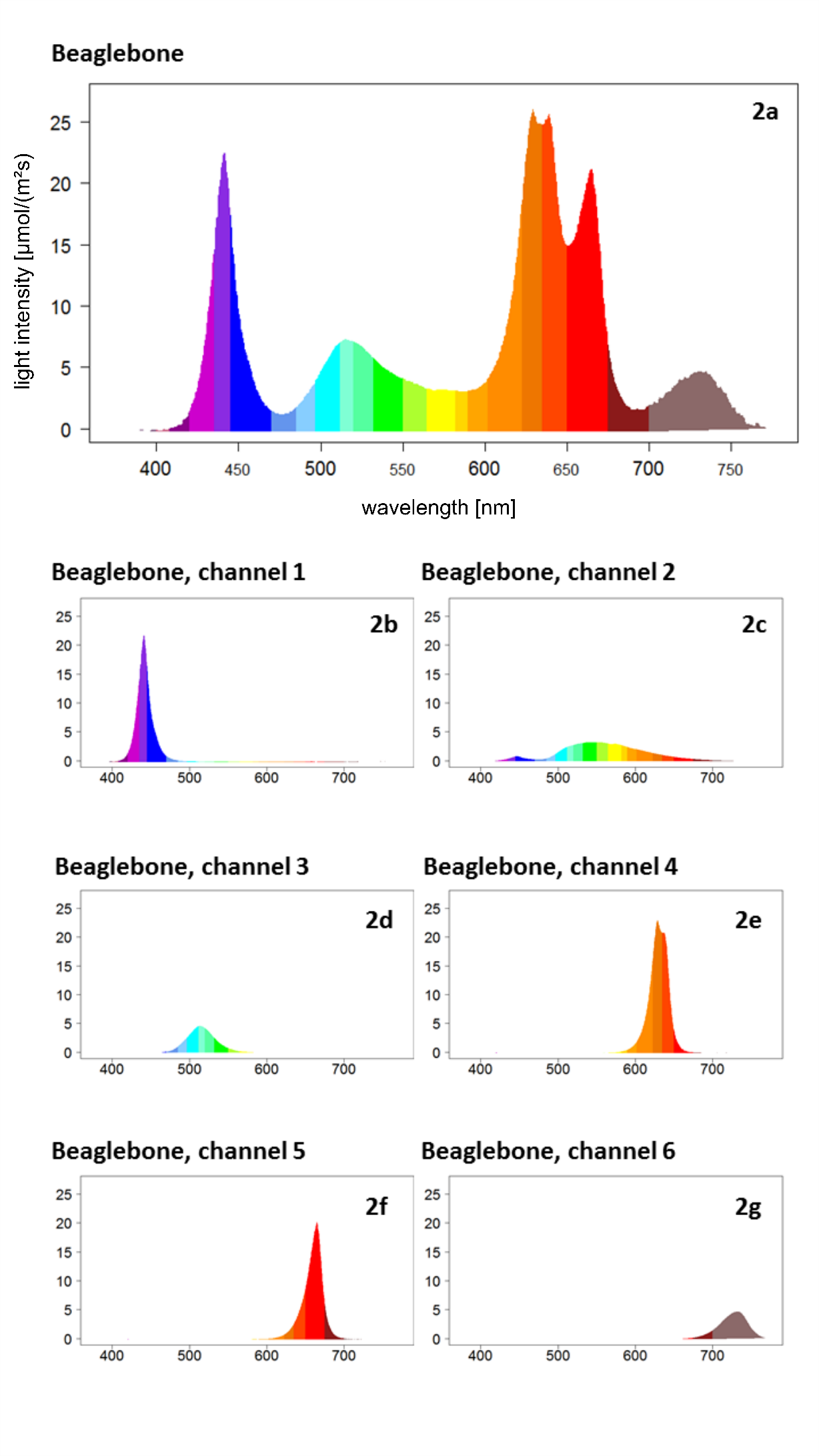


**Fig. S2.2** Emission spectrum of LED chamber type “Beaglebone”. **a** All channels at 100% intensity combined, **b-g** single channels at 100%. Numbers 2a – 2g in the top right corner refer to Table S4.


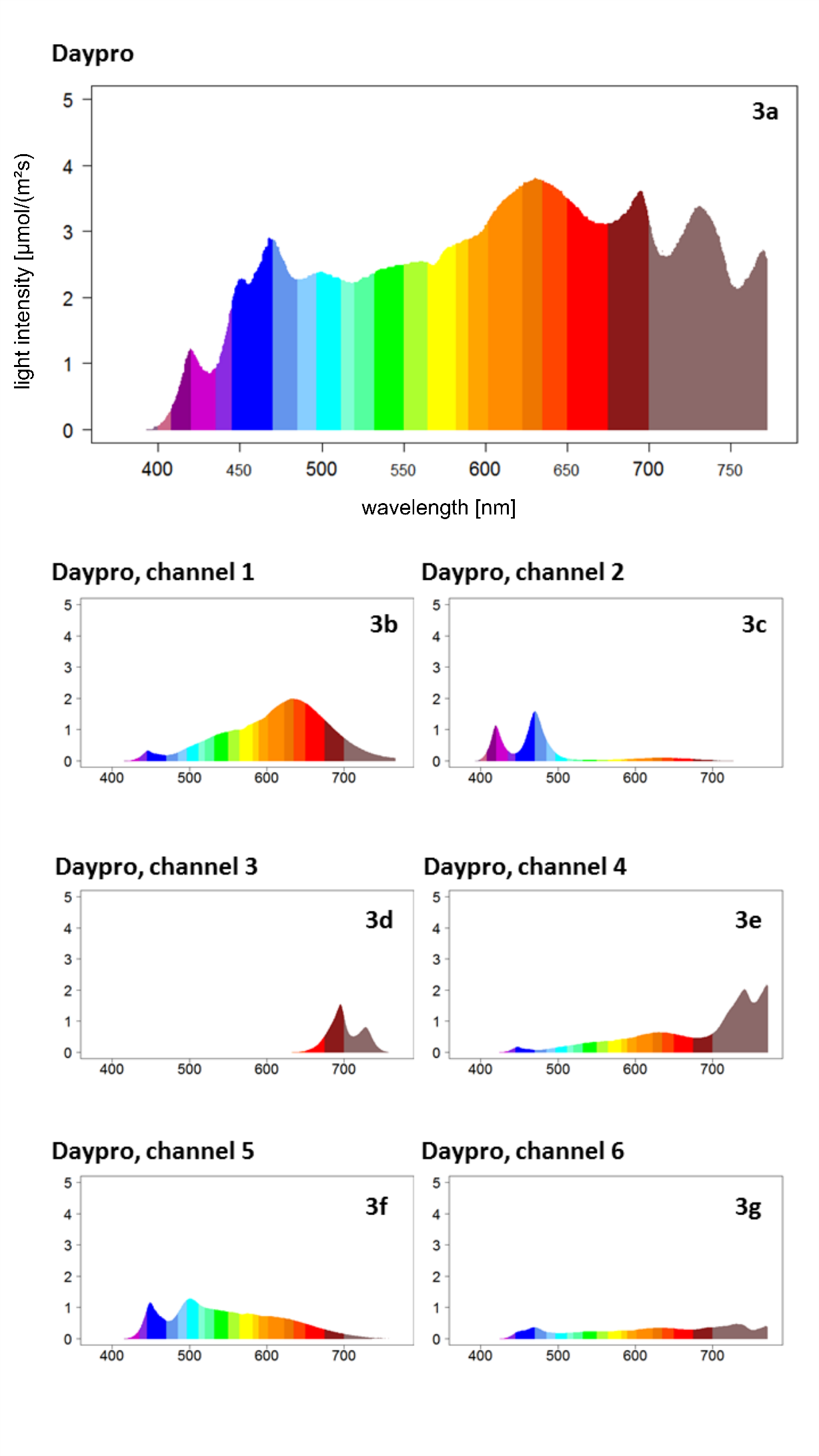


**Fig. S2.3** Emission spectrum of LED chamber type “daytune”. **a** All channels at 100% intensity combined, **b-g** single channels at 100%. Numbers 3a – 3g in the top right corner refer to Table S4.


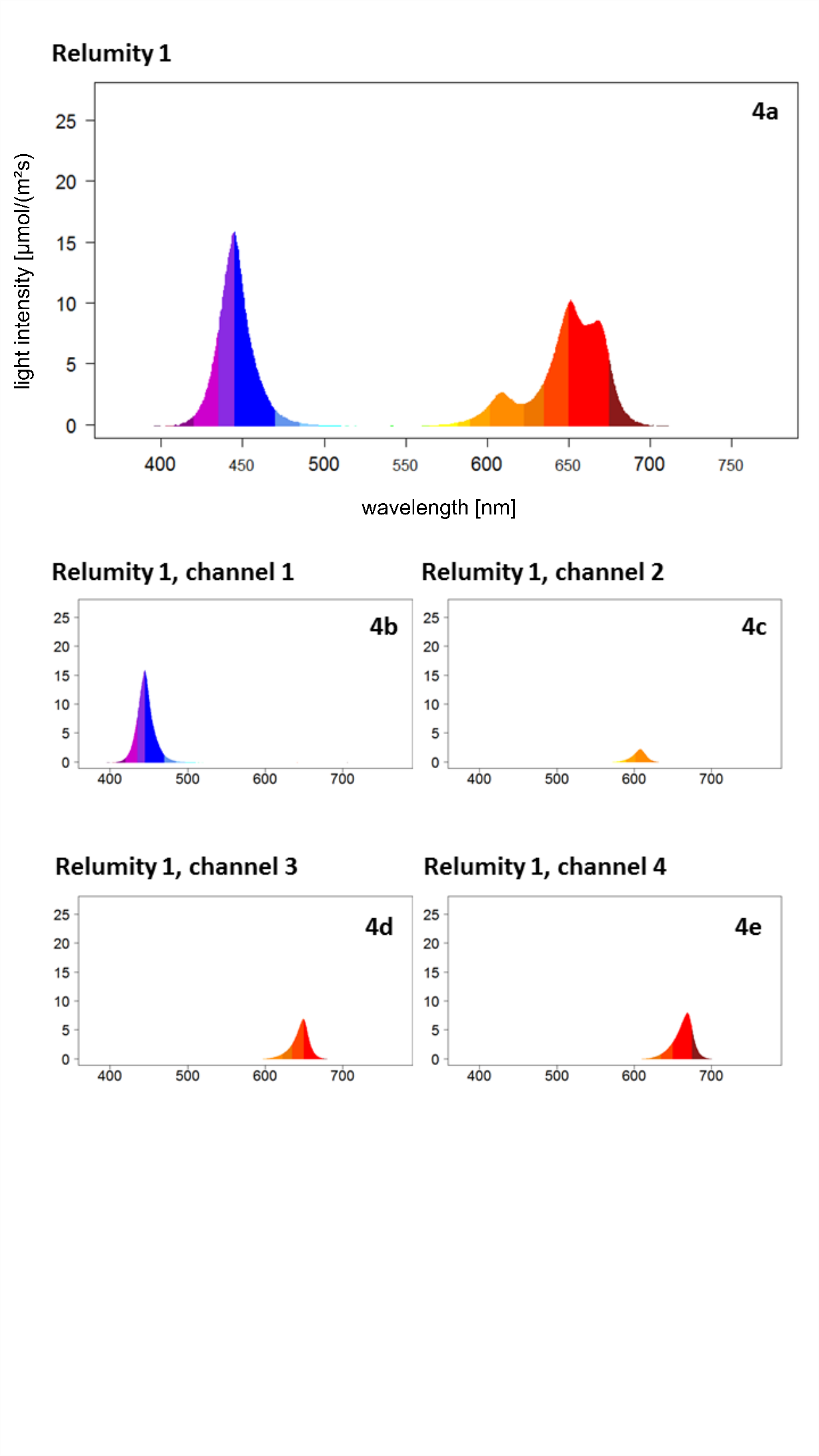


**Fig. S2.4** Emission spectrum of LED chamber type “Relumity” n°1. **a** All channels at 100% intensity combined, **b-e** single channels at 100%. Numbers 4a – 4e in the top right corner refer to Table S4.


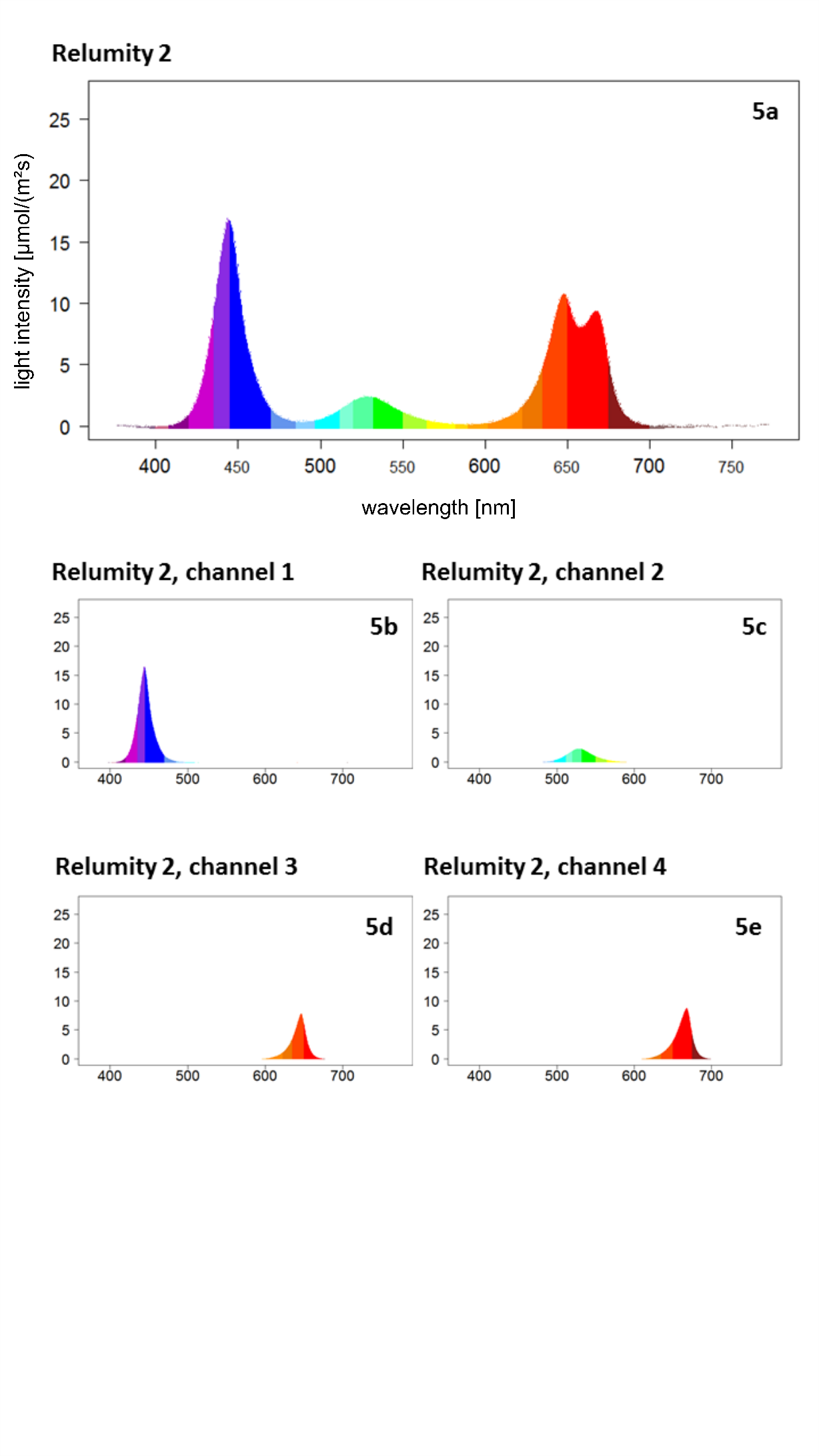


**Fig. S2.5.** Emission spectrum of LED chamber type “Relumity” n°2. **a** All channels at 100% intensity combined, **b-e** single channels at 100%. Numbers 5a – 5e in the top right corner refer to Table S4.


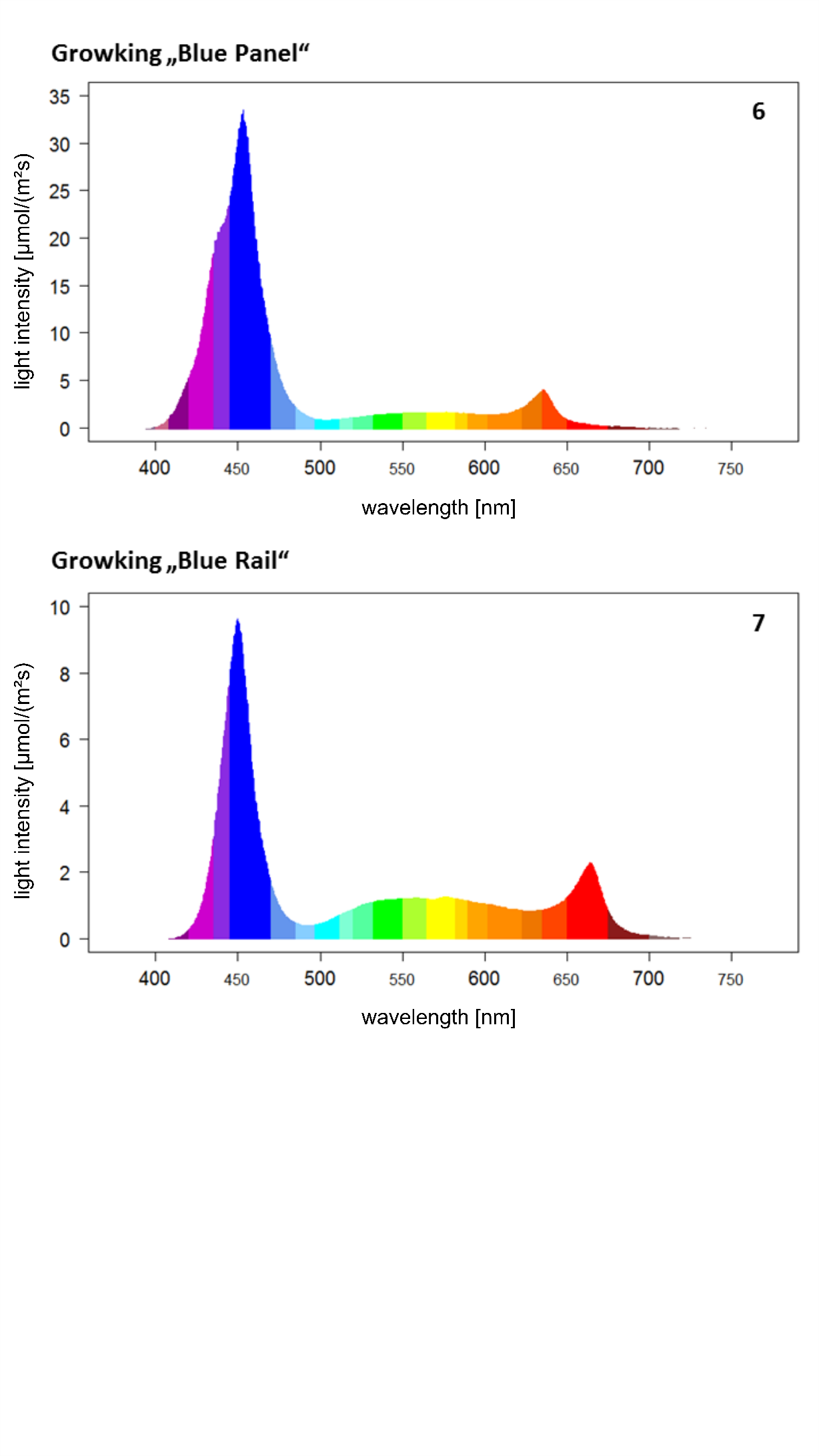


**Fig. S2.6.** Emission spectra of LED chamber type “Growking Blue Panel” (top) and “Growking Blue Rail” (bottom) at 100% intensity. Numbers 6 and 7 in the top right corner refer to Table S4.


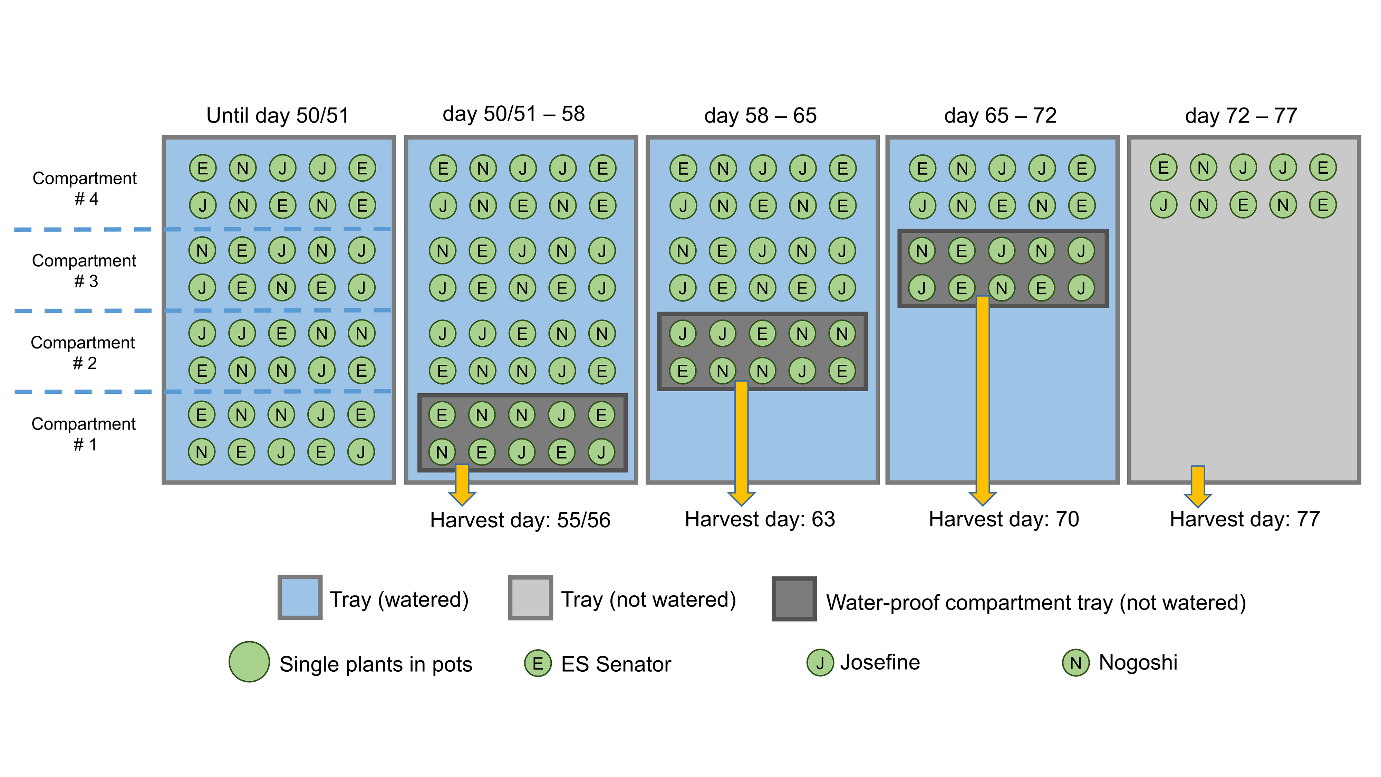


**Fig. S3.** Harvesting scheme for staggered harvest time experiments 52 and 60 (Table S6).


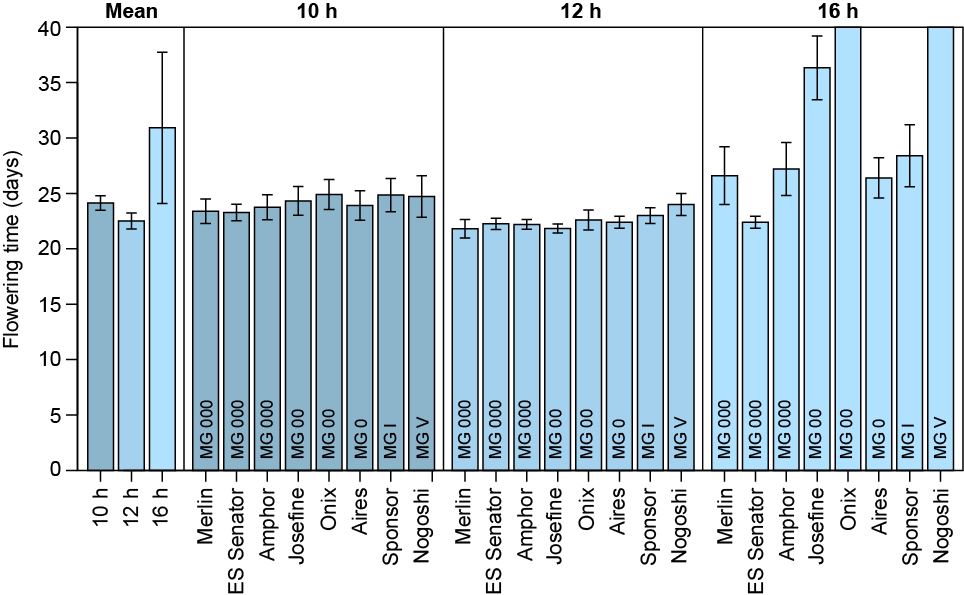


**Fig. S4** Barplot for mean flowering time of the eight soybean cultivars exposed to different day lengths. Soybean maturity group is indicated for each genotype; light conditions: red (<670nm), green and blue light at 555 µmol/(m²s).


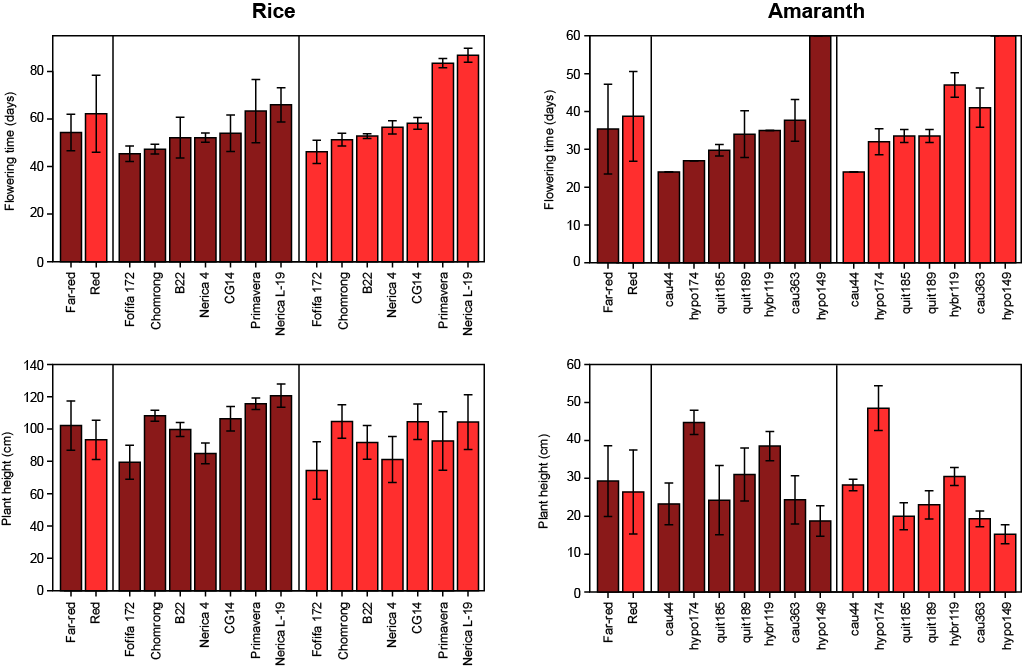


**Fig. S5** Flowering time and plant height of seven rice and amaranth genotypes under red and far-red light recipe treatments shown across all genotypes and for each genotype separately.


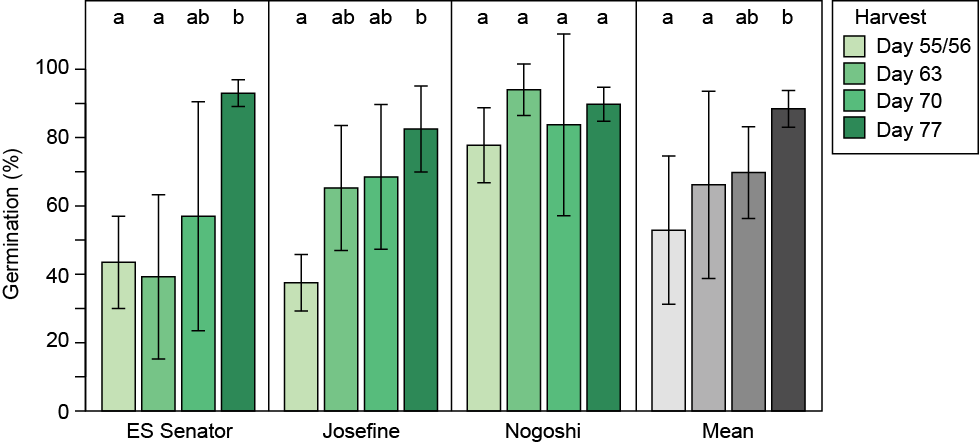


**Fig. S6** Mean germination of three soybean genotypes separately and the means across the genotypes for the staggered harvest time (Experiments 52 and 60; Table S6). Different letters on top of the bars indicate significant differences of the means within the genotype and the overall means, respectively, according to TukeyHSD test (α=0.05).


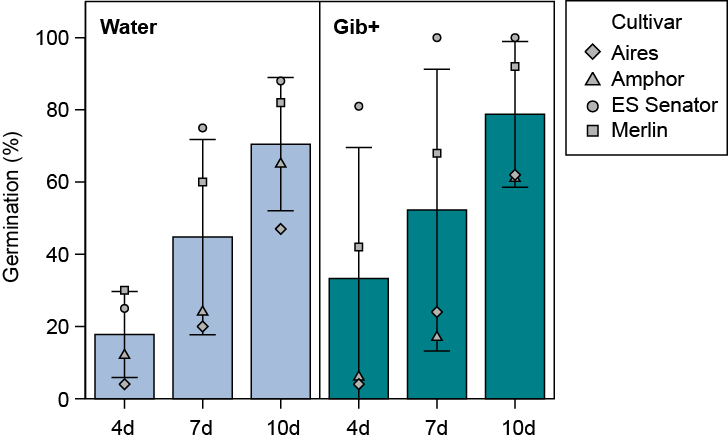


**Fig. S7** Mean germination on day 4, 7 and 10 of seed from 56 day old soybean plants treated with Gibb+ solution and water as a control. Grey symbols indicate germination of individual genotypes.


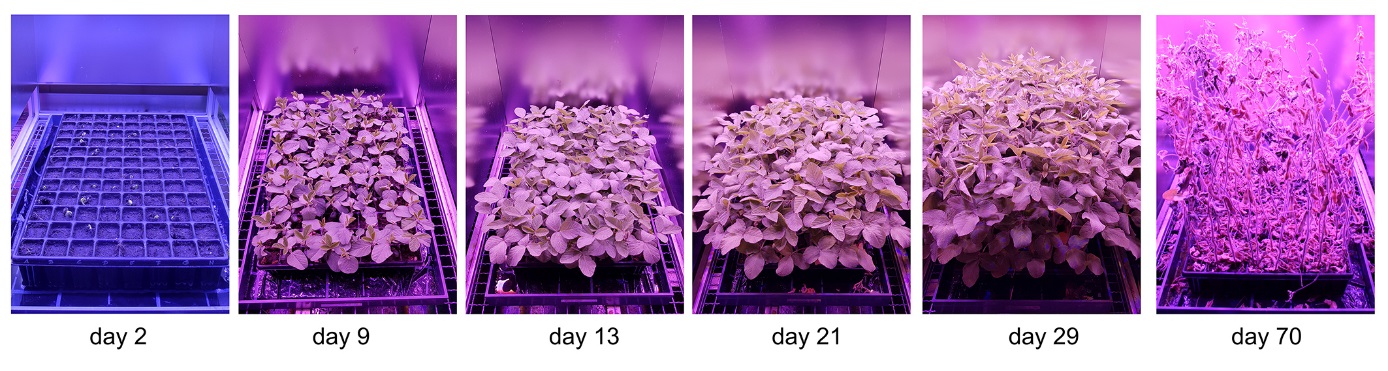


**Fig. S8** Time lapse of soybeans grown in 96-cell trays with 16 cm² each cell and 8 cm height (75 cm³ each cell).
